# Acute Effects of Dropsets Among Different Resistance Training Methods in Upper Body Performance

**DOI:** 10.2478/v10078-012-0069-6

**Published:** 2012-10-23

**Authors:** Claudio Melibeu Bentes, Roberto Simão, Travis Bunker, Matthew R. Rhea, Humberto Miranda, Thiago Matassoli Gomes, Jefferson Da Silva Novaes

**Affiliations:** 1 Universidade Federal do Rio de Janeiro. Programa de pós-graduação stricto-sensu EEFD/UFRJ. Rio de Janeiro, BRASIL.; 2 A.T. Still University, Human Movement Program, Mesa, Arizona, USA.; 3 RACE Rx Academy of Exercise Sciences, Logan, Utah, USA.

**Keywords:** Performance, resistance exercise, number of repetitions, training method

## Abstract

The aim of this study was to compare the influence of including dropset exercises in different orders, both in the pre-exhaustion, as in the post-exhaustion method, and to analyze the performance of total work on the bench press and chest flying exercise. Twenty-two male volunteers with a recreational experience in ST were evaluated in six visits in non-consecutive days, at approximately the same time of the day. During the first visit, subjects signed an informed consent form and underwent an anthropometric evaluation and testing of 10RM. The second visit involved a re-test of 10RM. From third to sixth visits, the subjects were randomly grouped into the following experimental situations: 3rd Visit (V3 – Post-exhaustion): Bench Press (dropset) + Chest Flying (10RM); 4th visit (V4 - Post-exhaustion): Bench Press (10RM) + Chest Flying (dropset); 5th Visit (V5 - pre-exhaustion): Chest Flying (dropset) + Bench Press (10RM); 6th Visit (V6 - pre-exhaustion): Chest Flying (10RM) + Bench Press (dropset). The protocol of dropset was performed with 3 sets and no rest intervals 10RM + 80% 10RM + 60% 10RM. An interval between sets was adopted for 2 minutes. The primary results showed a significant difference in Total Work for visits V3 and V6, which was included in the dropset multiarticular exercises. These results suggest that the exercise order with the dropset method in the pre-exhaustion or post-exhaustion methods had an acute influence on Total Work.

## Introduction

Increasing muscle strength is generally deemed as an important consideration for training programs with the purpose to improve performance, general health, and physical fitness (ACSM, 2011; ACSM, 2006). The paramount activity to promote increases in muscle strength is strength training and optimally designed training programs which are based on scientific principles that govern the prescription of different training variables. This is normally achieved by manipulating volume, exercise order, intensity, exercise type, between-set rest intervals, and other variables (ACSM, 2009; ACSM, 2011). However, some strength training methods used in conditioning centers require more research. Several methods that are commonly used by fitness practitioners include pre-exhaustion (PRE) and dropset (DS) yet the influences of such techniques are relatively unknown.

In scientific strength training literature (Augustsson et al., 2003; Gentil et al., 2007), the rationale for the PRE method utilization may be an increase of motor unit recruitment during fatigue, resulting in greater muscle activation for subsequent multi-joint exercise (Gentil et al., 2007; Brennecke et al., 2009; Da Silva et al., 2010). But the evidence is contradictory regarding the practical application of this method, both related to training and rehabilitation (Augustsson et al., 2003; Brennecke et al., 2009; Da Silva et al., 2010; Fleck and Kraemer, 2006; Simão et al, 2012). The DS method, as discussed in nonscientific strength training literature, may enable greater amounts of muscular work in higher intensities by providing short rest periods between work bouts. Augustsson et al. (2003) observed a decrease in electromyography amplitude of the quadriceps muscle during leg press exercise with PRE compared to without PRE in the leg press exercise. Gentil et al. (2007) investigated the effects of PRE on upper-body muscle activation during the bench press exercise and reported that the peck deck exercise, when performed immediately before the bench press exercise leads to similar electromyography amplitude of the anterior deltoid and the pectoralis major muscles. However, they observed an increase in the triceps brachii activation with the worst performance during the bench press exercise with PRE. Despite the decrease in performance, this increase in electromyography intensity during PRE may also be altered because fatigue of some muscles can be compensated by increasing motor unit recruitment of other muscles in an attempt to maintain the required performance. Brennecke et al. (2009) investigated the effects of PRE on upper-body muscle activity during the bench press in trained subjects finding that PRE did not affect the temporal pattern of muscular activity and muscular unit recruitment of the pectoralis major or anterior deltoid muscles. There was a related increase in surface electromyography signal amplitude of triceps brachii muscle during the bench press. However, little is known about the efficiency of dropset on strength performance. Gaps in the current literature surrounding the use of this methodology suggest the need to further examine its use and impact. The method of dropset combined with pre-exhaustion in the same training program requires further investigation so that their benefits and proper applications become clear. The purpose of the present study was to compare the influence of including dropset exercises in different orders in a strength training program, both in the method PRE, and in the post-exhaustion (POST) on the total work performed (calculated by multiplying total repetitions (RM) x workload (KG) during the bench press and the chest flying exercises.

## Material and Methods

### Participants

To compare the influence of including a dropset with pre-exhaustion and post-exhaustion on total work, subjects performed six visits, four visits to strength training sessions and two visits for 10RM loads determination for each exercise. The subjects were randomly assigned to particular sessions (Table 1). In the first day, and on the other three days, the procedures were exactly the same but with the other exercise sequences. The number of repetitions was measured in each session to determine the total amount of work performed based on each sequence.

Twenty-two men (age 22.5 ± 3.04 years, body height 176.36 ± 5.05 cm, body mass 75.32 ± 5.96 kg) performed a strength-training program for at least one year with a minimum training frequency of 3-times per week prior to the study. All subjects answered the Physical Activity Readiness Questionnaire and signed an informed consent form before participation. According to the Declaration of Helsinki, the following exclusion criteria were adopted: All subjects were accustomed to training with both exercise orders; None of the subjects had a recent history of significant upper-body injury; Before participation, each subject read and signed a detailed consent form; The Ethics Committee of Federal University of Rio de Janeiro approved the study protocol.

### Ten Repetition Maximum Testing (10RM)

To obtain reliable 10RM loads, data were assessed during 2 nonconsecutive days following the exercise sequence: Bench Press (BP) and Chest Fly Exercise (FE). During the 10RM test, each subject performed a maximum of three 10RM attempts for each exercise with 5-minute rest intervals between attempts. After the 10RM load in a specific exercise was determined, an interval not shorter than 10 minutes was allowed before the 10RM determination of the next exercise. Standard exercise techniques were followed for each exercise. A paired student t-test did not show significant differences between the 10RM tests for any of the exercises (p>0.05). The heaviest load achieved in both days was considered the 10RM.

### Strength Testing

The four strength-training sessions were performed on nonconsecutive days with at least 48 hours between experimental sessions. During the sessions, a load for maximal 10RM for both exercises was performed. For such, the session that involved a DS occurred with two consecutive decreases of twenty percent in maximal workload (10RM + 80%10RM + 60%10RM) immediately after the execution of 10RM, without rest interval between declines (Fleck and Kraemer, 2006). No pause was allowed between the eccentric and concentric phase of a repetition or between repetitions, and the exercise sequence was exactly the same for 10RM testing. During all strength-training sessions, subjects were asked not to perform a valsalva maneuver. Strength training sessions of individual subjects were performed at the same time of the day. All sets were performed until muscular failure or a negative change in lifting mechanics was observed. We adopted a 2-min rest interval between exercises, except on the DS. The number of repetition (RM) and workload was collected at the end of each set. Total Work (TW) was calculated by multiplying total repetitions (RM) x Workload (kg). From the third to the sixth visits, subjects were randomly grouped into the following experimental situations: 3rd Visit (V3 - POST): Bench Press (dropset) + Chest Flying (10RM); 4th visit (V4 - POST): Bench Press (10RM) + Chest Flying (dropset); 5th Visit (V5 - PRE): Chest Flying (dropset) + Bench Press (10RM); 6th Visit (V6 - PRE): Chest Flying (10RM) + Bench Press (dropset). All subjects were asked not to exercise any of the muscle groups involved in the test 48 hours before the test.

### Statistical Analyses

The statistical analysis was initially performed using the Shapiro–Wilk normality test and the homocedasticity test (Bartlett criterion). As mentioned, the 10RM tests were found to be similar when tested on two occasions prior to performing the different sequences. In addition the 10RM result was highly correlated as demonstrated by an Intraclass Correlation Coefficient (BP, r = 0.9843; FE, r = 0.9416). To compare a total work (Total RM x Workload) and the Total RM in different sequences, an ANOVA with repeated measures was performed. Specific differences were determined using the Tukey HSD post hoc test. An alpha level of p<0.05 was considered statistically significant for all comparisons. Additionally, to determine the magnitude of the findings, effect sizes (ESs; the difference between pretest and posttest scores divided by the pretest SD) were calculated for compare the different data visits, and the scale proposed by Rhea (Rhea et al., 2004) was used to determine the magnitude of the ES. All statistical analyses were carried out using SPSS statistical software package version 19.0 (SPSS Inc., Chicago, IL).

## Results

**Figure 1:**
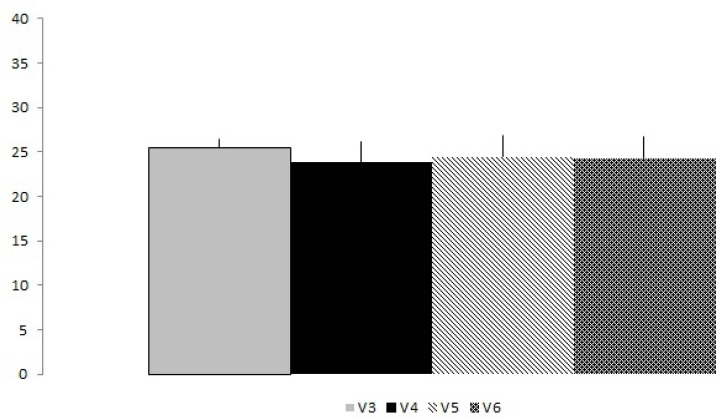
Means and standard deviation of the total repetitions of each experimental group (p≥0.05)

**Figure 2:**
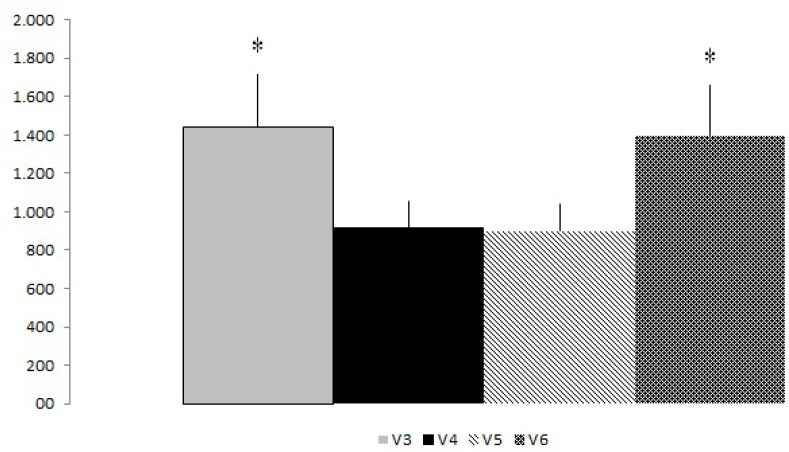
*Total Work for each Experimental Approach (mean ± SD)*. *Significant differences between protocols for V3 x V4; V4 x V6; V5 x V6; V3 x V5 (p < 0.05)

The total number of RM showed no significant difference between experimental groups (Figure 1). Descriptive data are presented in Figure 1. The mean of each visit compared post-exhaustion and pre-exhaustion method with or without dropset and also showed no significant differences. However, when the mean of the total work (total repetitions (RM) x workload (kg) (Figure 2) was compared, significant differences were found for groups V3 and V6 (p < 0.05). Table 2 shows the total work effect size mean for each experimental approach. The results showed large effect size values between protocols V3 x V4; V4 x V6; V5 x V6; V3 x V5.

## Discussion

The major finding of the present study showed that for groups in which there was an inclusion of a dropset in the bench press (V3 and V6), regardless of the order applied, the total work was affected, with a significant difference compared to the dropset in the chest flying exercise (V4 and V5). Furthermore, the ES data demonstrated higher magnitudes at suggesting that the inclusion of a dropset in the bench press produces more total work. The groups V3 and V6 had the largest number of results compared to the V4 and V5 of the total work (p < 0.05). Such result may have occurred because of the characteristics of multi-joint exercise. According to some authors in similar studies that have investigated during the pre-exhaustion, the hypotheses for the maintenance of exercise performance is the inclusion of other muscles involved in exercise, especially multi-joint exercises (Brennecke et al., 2009; Fleck and Kraemer, 2006; Simão et al., 2007).

This seems to be the first study dedicated to verifying the inclusion of dropset in the pre-exhaustion method. In the analysis of results, it was interesting to notice that there were no significant differences between visits V3, V4, V5 and V6 regarding averages of repetitions and total repetitions, indicating that the major factor was the workload or total work, as the variable that changed absolutely.

The dropset method has been included within the two different exercise orders of the post-exhaustion and pre-exhaustion. In recent studies, results show that if an exercise is placed at the beginning of a training session, more repetitions can be performed, generating a higher total work (Augustsson et al., 2003, Kraemer and Ratamess, 2004; Simão et al., 2005). In this study, V3 and V6 groups, in which there was the inclusion of a dropset in the exercise of bench press, there was a higher total work compared to groups V4 and V5 (p < 0.05). According to the results of this study, the exercise order was not the main factor to affect the performance of total work but potentially the type of exercise (multi-joint) being tested. The hypothesis was that the performance of dropset in the chest flying exercise (V4 and V5) would increase the fatigue in the pectoralis major muscle and therefore a decrease in performance of total work (p the > 0.05). As for the combination of the methods of pre-exhaustion with dropset in the bench press, there was a significant difference comparing groups V6 and V3 with dropset in BP.

Some studies that investigated an acute effect in different exercise order (Augustsson et al., 2003; Brennecke et al., 2009; Fleck and Kraemer, 2006) have a methodology that allows some comparisons to the current study. More recently, Gentil et al. (2007) investigated the effect of PRE in the upper body by analyzing EMG in the pectoralis major, anterior deltoid and triceps brachii during the performance of the total number of repetitions and total work. The study was performed with thirteen trained men and the results showed a significant difference for the first exercises of both sets of pre-exhaustion. However, there were no significant differences for total work and the total repetitions of the exercises. These results differ from our findings, where the total work was higher for the groups V3 and V6, with the performance of a dropset in the bench press, independent of the exercise order. However, our results demonstrated that the inclusion of the dropset in multi-joint exercise may have promoted an increased performance. The dropset method may have optimized the neuromuscular responses and prolonged neuromuscular activity (Brennecke et al., 2009; Da Silva et al., 2010).

Augustsson et al. (2003), with a methodology applied to the lower body, obtained results that converged with Gentil et al. (2007), specifically for leg press and leg extension. The protocol was applied in 17 trained men with measures of maximum repetitions, total work and electromyography of the rectus femoris, vastus lateralis and the gluteus maximus. The results showed higher scores for number of repetitions when the pre-exhaustion was not used. Nevertheless, these results differ from our findings, where the total repetitions showed no significant differences between groups and the total work was significantly different between groups with a dropset in the multi-joint exercise. However, in the Augustsson et al.’s study, the experiments were done in the lower limbs where muscle volume is higher (Kraemer and Ratamess, 2004; Kraemer et al., 1997; Simão et al., 2005; Simão et al., 2007).

The dropset method in both pre-exhaustion or in post-exhaustion may have promoted better scores when done in multi-joint exercise, regardless of the order in which it was employed. However, this method of training needs further examination to justify its use as a training method. It is suggested that future research investigate muscle activity and hormonal adaptations when employing pre-exhaustion and dropset methods to further assist in the identification of the value and proper implementation of these training methods.

## Conclusions and practical implications

From the present data, it can be concluded that a combination of pre-exhaustion with dropset in a strength training session involving the bench press exercise is capable of generating a greater total work when compared to a chest flying exercise. The dropset method in both pre-exhaustion or in post-exhaustion may promote higher total work when done in multi-joint exercise, regardless of the order in which it was employed. Dropset procedures may prove valuable in attempts to increase total work during a training session when increased strength or hypertrophy is the goal.

## Figures and Tables

**Table 1:** Training Sessions

V3	Bench Press (dropset)	Chest Fly Exercise (10RM)
V4	Bench Press (10RM)	Chest Fly Exercise (dropset)
V5	Chest Fly Exercise (dropset)	Bench Press (10RM)
V6	Chest Fly Exercise (10RM)	Bench Press (dropset)

Dropset Method (10RM +80%10RM + 60%10RM) without interval

**Table 2:** Total work effect size mean for each experimental approach

	**V3 x V4**	**V3 x V5**	**V3 x V6**	**V4 x V5**	**V4 x V6**	**V5 x V6**
ES	1.881	1.946	0.154	0.130	3.445	3.443
Magnitude	***Large***	***Large***	*Trivial*	*Trivial*	***Large***	***Large***

Large effect size between protocols V3 x V4; V3 x V5, V4 x V6; V5 x V6
